# Structural-Functional Analysis Reveals a Specific Domain Organization in Family GH20 Hexosaminidases

**DOI:** 10.1371/journal.pone.0128075

**Published:** 2015-05-29

**Authors:** Cristina Val-Cid, Xevi Biarnés, Magda Faijes, Antoni Planas

**Affiliations:** Laboratory of Biochemistry, Institut Químic de Sarrià, Universitat Ramon Llull, Barcelona, Spain; University of Copenhagen, DENMARK

## Abstract

Hexosaminidases are involved in important biological processes catalyzing the hydrolysis of N-acetyl-hexosaminyl residues in glycosaminoglycans and glycoconjugates. The GH20 enzymes present diverse domain organizations for which we propose two minimal model architectures: Model A containing at least a non-catalytic GH20b domain and the catalytic one (GH20) always accompanied with an extra α-helix (GH20b-GH20-α), and Model B with only the catalytic GH20 domain. The large *Bifidobacterium bifidum* lacto-N-biosidase was used as a model protein to evaluate the minimal functional unit due to its interest and structural complexity. By expressing different truncated forms of this enzyme, we show that Model A architectures cannot be reduced to Model B. In particular, there are two structural requirements general to GH20 enzymes with Model A architecture. First, the non-catalytic domain GH20b at the N-terminus of the catalytic GH20 domain is required for expression and seems to stabilize it. Second, the substrate-binding cavity at the GH20 domain always involves a remote element provided by a long loop from the catalytic domain itself or, when this loop is short, by an element from another domain of the multidomain structure or from the dimeric partner. Particularly, the lacto-N-biosidase requires GH20b and the lectin-like domain at the N- and C-termini of the catalytic GH20 domain to be fully soluble and functional. The lectin domain provides this remote element to the active site. We demonstrate restoration of activity of the inactive GH20b-GH20-α construct (model A architecture) by a complementation assay with the lectin-like domain. The engineering of minimal functional units of multidomain GH20 enzymes must consider these structural requirements.

## Introduction

N-acetyl-β-hexosaminidases are widely distributed in nature participating in many biological processes [1,2]. They catalyze the hydrolysis of N-acetyl-hexosaminyl residues in glycoproteins, glycosaminoglycans, chitin, and glycolipids, and are classified in CAZY families GH3, GH20 and GH84 (Carbohydrate Active Enzymes database, www.cazy.org [3]). GH20 hexosaminidases are found in crustaceans, insects and fungi, participating in the degradation of chitin, and in bacteria, where they are involved in the catabolism of oligosaccharides that serve as nitrogen and carbon sources. From the biotechnological point of view, hexosaminidases, together with chitinases, are important enzymes for industrial chitin degradation, the most abundant polysaccharide after cellulose [2]. They are also powerful insect and fungal control agents as biopesticides. In humans, the two lysosomal hexosaminidases HexA and HexB are responsible for the hydrolysis of GlcNAc and GalNAc residues of glycosphingolipids such as ganglioside GM2 and their dysfunction is linked to neurodegenerative disorders known as Tay-Sachs and Sandhoff diseases [4,5]. In addition, some bacterial hexosaminidases contribute to the virulence of some infections in humans as in the case of *Actinobacillus actinomycetemcomitans* that colonizes the human oral cavity and causes periodontitis and *Streptococcus pneumoniae* infections [2].

GH20 enzymes comprise not only β-N-acetylhexosaminidases (EC.3.2.1.52), catalyzing the removal of terminal non-reducing N-acetylhexosamine residues, but also lacto-N-biosidases (EC.3.2.1.140), hydrolyzing the terminal lacto-N-biosyl residues from the non-reducing end of oligosaccharides such as human milk oligosaccharides (HMOs). HMOs play an important role in the development of intestinal flora and in the modulation of the immune system of breast-fed infants [6–8]. HMOs are constituted by two core tetrasaccharide structures: the lacto-N-tetraose (type 1) characterized by lacto-N-biose (Galβ1,3GlcNAc) and lactose units linked by a β-1,3 glycosidic bond, and lacto-neo-N-tetraose (type 2) formed by β-1,3-linked N-acetyllactosamine (Galβ1,4GlcNAc) and lactose. They are elongated to different isomers of lacto-N-hexa-, octa- and decaose and then, decorated with fucose and sialic residues via α-1,2/3/4 linkages and α-2,3/6 respectively [9,10]. Bifidobacteria have a unique metabolism on HMO resulting in their predominant proliferation in the colon of breast-fed infants [7]. Specifically, *Bifidobacterium bifidum* metabolizes type 1 human milk oligosaccharides by the action of extracellular sialidases, fucosidases and lacto-N-biosidase.

**Fig 1:**
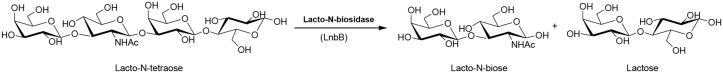
Hydrolytic reaction of lacto-N-tetraose catalyzed by *B*. *bifidum* lacto-N-biosidase.

To date, the structures of thirteen GH20 enzymes are known. Their domain organization is diverse and several accompanying domains are present apart from the catalytic GH20 domain. In addition, some of them exist as dimeric proteins. We aimed at defining the minimal functional domain architecture for enzymatic activity in these GH20 family enzymes. We chose the large *B*. *bifidum* lacto-N-biosidase (LnbB) as a model protein because of its complex domain organization among GH20 enzymes and its significance in the metabolism of human milk oligosaccharides. The LnbB gene encodes for a membrane-anchored extracellular enzyme of 1112 amino acid residues. Recently, it was cloned and the recombinant full length protein was characterized confirming that this enzyme catalyzes the hydrolysis of the tetrasaccharide Galβ1,3GlcNAcβ1,3Galβ1,4Glc to lacto-N-biose (Galβ1,3GlcNAc) and lactose (Fig 1) [11,12]. LnbB is a retaining exo-enzyme that acts by substrate-assisted catalysis, which is a common feature of GH20 enzymes.

By means of structural and sequence analysis of GH20 enzymes of known structure, we have established two levels of domains organization in this family, and further engineered LnbB in different truncated forms according to these models. We report the structural requirements for functionality, of this and the other GH20 enzymes, to obtain a minimal functional unit that retains the enzymatic activities. The importance of a remote element in the active site such as loop 2 seen in some GH20 enzymes is analyzed in all structures. It is shown that the isolated GH20 catalytic domain in LnbB is inactive and requires a remote element provided by the adjacent lectin domain for activity, as observed in a complementation experiment. This structural and functional analysis provides new insights for further enzyme engineering for biotechnological and biomedical applications.

## Materials and Methods

### Cloning, expression and purification of Lacto-N-biosidase enzyme *from Escherichia coli*

Two synthetic genes of Lacto-N-biosidase from *Bifidobacterium bifidum* (LnbB), codon-optimized for the expression in *Escherichia coli* were produced by GenScript (GenScript, NJ, USA). The LnbB gene (37–1064) was cloned into the pET24b plasmid (Novagen, Madison, WI, USA) using the restriction sites NdeI and XhoI, and the construct A gene (40–528) into the pET28a+ vector using NdeI and Bpu1102. Constructs B (176–497) and Bα (176–528) genes were obtained by PCR reaction using construct A as template, and constructs D (40–776) and F (546–776) genes using the full length LnbB gene. The following forward and reverse primers were used (restriction site are underlined): P1 (5’-GGAATTCCATATGAAGCCGAAATATAAAGAACGT-3’) and P2 (5’-TAATAGCTCAGCGGCGTCAAC-3’) for construct B; P1 and P3 (5’-TAATAGCTCAGCACGGGAGTCACTCCA-3’) for construct Bα; P4 (5’-GCGTATCCATGGCTGATGACTCCGCAGCAGGCTA-3’) and P5 (5’-GCGTATCTCGAGCAGGCTGCCGGTCAGC-3’) for construct D, and P6 (5’-GCGTATCCATGGTGGATGCGGGTATC-3’) and P5 for construct F. All constructs were cloned into the pET28a+ vector. *E*. *coli* DH5 cells were transformed and positive transformants were verified by DNA sequencing.

Expression and purification of the full length protein and constructs were carried out as follows: recombinant cultures of *E*. *coli* BL21 (DE3) *star* cells were grown following an autoinduction protocol [13] in LB medium with 30 μg·mL^-1^ kanamycin, 25mM Na_2_HPO4, 25mM K_2_HPO4, 50mM NH_4_Cl, 5mM Na_2_SO_4_, 2mM MgSO4, 0.5% glycerol, 0.05% glucose and 0.2% lactose at 250 rpm and 30°C for 24 h. The full length protein and constructs A, B and Bα were also obtained using 1 mM IPTG induction after 16 h of growth at 37°C in LB medium with kanamycin achieving similar results. Cells were harvested by centrifugation (4000*g*, 15 min, 4°C), resuspended in buffer A (Na_2_HPO_4_ 20mM, NaCl 150mM, pH 7.5) and disrupted in a cell disrupter (20000 psi). After centrifugation (20000*g*, 4°C, 2 h), the supernatant was loaded into a 1mL Ni^2+^-charged HiTrap chelating column chromatography. Proteins were purified to homogeneity by a linear gradient of Buffer B (Na_2_HPO_4_ 20 mM, NaCl 150 mM, imidazole 500mM, pH 7.5). Collected fractions were analyzed by SDS-PAGE and fractions containing the proteins were combined and dialyzed against buffer A with a 30 kDa cut-off membrane. All proteins were purified further using an XK16/60 Superdex 200 column (GE Healthcare), preequilibrated with buffer A. Fractions were pooled, concentrated with a Centricon YM-10 unit (Merk Millipore) and stored at 4°C. Protein concentrations were determined spectrophotometrically by the BCA method measuring A590 nm using BSA as standard [14].

Protein size was determined using dynamic light scattering in a Nano ZS Nanosizer (Malvern Instruments Ltd., UK) with a laser light wavelength of 632.8 nm and a scattering angle of 173 degrees. Temperature was set at 25°C. Protein solutions in buffer A were measured without previous dilution. Full length protein showed a single peak for monodisperse particles of 10 nm and PDI 0.30, which is consistent with monomeric form.

### Kinetics of the hydrolase activity

Lacto-N-biosidase activity assays with *p*-nitrophenyl β-lacto-N-bioside (Toronto Research Chemicals Inc.) were performed at 30°C in a volume of 0.1 mL using 96-wells microtiter plates measuring p-nitrophenol concentration at 405 nm. Enzymatic reactions were performed in a reaction mixture containing 0.25 mM substrate and 50mM of citrate-phosphate buffer (pH 4.5). After pre-incubation for 5 min, reactions were initiated by addition of the enzyme (20 nM of full length protein and construct D, 20 nM-4 μM of construct A). At regular time intervals, reactions were quenched by the addition of 0.15 mL of quenching buffer (0.5 M of glycine, pH 10) and p-nitrophenol release was measured by absorbance at 400 nm with a microplate-reader MRX (Cultek). Triplicate measurements at each concentration were performed.

### Complementation assays

Enzymatic reactions with constructs A and F were performed using standard conditions in a Bravo liquid handling Robot (Agilent). After pre-incubation of construct A (50 nM) and F (25 nM-500 nM) for 5 minutes, addition of the substrate initiated the reaction. Triplicate measurements at each concentration were performed. The complex of constructs A and F was also characterized using gel filtration chromatography. The mixture of construct A (42 μM) with excess of construct F (245 μM) was loaded to the Superdex 200 column pre-equilibrated with buffer A and calibrated with protein standards. Fractions were further analyzed by SDS-PAGE and their hydrolase activity was determined. This purification was performed by duplicate.

Enzymatic activities of construct A (50 nM) in the presence of BSA at 1:1, 1:4 and 1:10 ratio were analyzed using standard conditions.

### GH20 structures and sequences retrieval and analysis

The list of currently available 3D structures of GH20 enzymes was retrieved from CAZY database (http://www.cazy.org, [3]). Protein structures were downloaded from the Protein Data Bank (http://www.pdb.org). Protein sequences were retrieved from UniProt (http://www.uniprot.org). Assignment of protein domains were obtained from different databases (Pfam, Superfamily, SCOP, PSI-BLAST and CAZY) and the consensus domain organizations are reported in Table 1. Protein structures were analysed and visualized with VMD [15]. Structure alignment was performed with MultiSeq [16] restricting the alignment region to GH20b and GH20 domains only. Sequence alignment was performed with PROMALS [17] with default options. Only the sequence fragments covering GH20b, GH20 domains and extra α-helix were considered.

**Table 1:** Domain organization of GH20 β-N-acetylhexosaminidases.

Model A					
Enzyme	Organism	Length (aa)	PDB	Domains^1^	GH20b-GH20-α location
**Monomeric**					
β-N-acetylhexosaminidase (SpHex)	*Streptomyces plicatus*	506	1HP5[A]	GH20b-GH20-α	19–494
β-N-acetylhexosaminidase (ScHex)	*Streptomyces coelicolor* A3(2)	535	4C7G[A]	GH20b-GH20-α	11–511
β-N-acetylhexosaminidase (Hex1T)	*Paenibacillus* sp.	978	3GH5[A]	GH20b-GH20-α-Lectin-CBD	15–493
Chitobiase (SmCHB)	*Serratia marcescens*	885	1QBB[A]	CHB_HEX-GH20b-GH20-α-CHB_HEX_C	215–814
Lacto-N-biosidase (LnbB)	*Bifidobacterium bifidum*	1112	4JAW[A,B]	GH20b-GH20-α-Lectin-CBM32-Ig like	34–515
β-N-acetylhexosaminidase (NahA)*	*Arthrobacter aurescens*	540	3RCN[A]	GH20b-GH20-	5–506
β-N-acetylhexosaminidase (Bf3009*)	*Bacteroides fragilis*	518	4PYS[A,B]	GH20b-GH20-α	21–498
**Heterodimeric**					
β-N-acetylhexosaminidase (HexA)	*Homo sapiens*	529/556	2GK1 [A,B,C,D,E,F,G]	GH20b-GH20-α	α:23–510 β:56–540
**Homodimeric**					
β-N-acetylhexosaminidase (HexB)	*Homo sapiens*	556/556	1NP0[A,B]	GH20b-GH20-α	56–540
β-N-acetylhexosaminidase (OfHex1)	*Ostrinia furnacalis*	593/593	3OZO[A]	GH20b-GH20-α	65–574
β-N-acetylhexosaminidase (GcnA)	*Streptococcus gordonii*	627/627	2EPN[A,B]	GH20b-GH20-α-Domain III	2–414
**Model B**					
**Enzyme**	**Organism**	**Length (aa)**	**PDB**	**Domains** ^1^	**GH20 domain location**
**Monomeric**					
β-1,6-N acetylglucosaminidase Dispersin B (DspB)	*Aggregatibacter actinomycetemcomitans*	361	1YHT[A]	GH20	20–342
β-N-acetylhexosaminidase (StrH)	*Streptococcus pneumoniae* R6	1312	3RPM[A,B]	GH20-GH20-G5-G5	190–538 635–972
	*Streptococcus pneumoniae TIGR4*	1312	2YL8[A]	GH20-GH20-G5-G5	190–538
			2YL9[A,B,C,D]	GH20-GH20-G5-G5	635–972

Data obtained using integration from different databases (Pfam, Superfamily, SCOP, PSI-BLAST and CAZY databases). PDB structure accession code and chain identifier used for structural analysis are indicated and domains actually present in the PDB structure are underlined.

^1^ **Domains**: domain nomenclature according to the consensus domain organization by integrating Pfam, Superfamily, SCOP, PSI-BLAST and CAZY databases. Preferentially CAZY annotation is used, if available. CBD: carbohydrate binding domain, CBM32: carbohydrate binding module family 32, CHB_HEX: chitobiose hexosaminidase accompanying domain in the N-terminus, CHB_HEX_C: chitobiose hexosaminidase C-terminal accompanying domain, Lectin: lectin-like domain according to PSI-BLAST, Ig like: Immunoglobulin-like fold domain, Domain III: C-terminal domain needed to dimerize according to authors, G5: accompanying domain of carbohydrate metabolism enzymes.

*: Oligomerization state in solution is unknown

## Results and Discussion

### 1. Structural domains organization of GH20 proteins

According to CAZY classification [3], GH20 family is mainly composed of both prokaryotic and eukaryotic β-N-acetylhexosaminidases with different substrate specificities. Using data integration from different databases (Pfam, Superfamily, SCOP, PSI-BLAST and CAZY databases), we report in Table 1 the consensus domains organization in GH20 proteins of known structure. The catalytic activity in this family of enzymes is assigned to the GH20 domain (PFAM code PF00728) which typically folds into a (β/α)_8_-barrel topology. Except for dispersin B from *A*. *actinomycetemcomitans* that presents only a single GH20 domain, the rest of structures show the catalytic domain accompanied by several domains with quite diverse functionalities: a non-catalytic domain, commonly named as GH20b, which is conserved in most GH20 enzymes although with unknown function, several lectin domains, carbohydrate binding domains, and other domains of unknown function.

Due to this diversity in domain organization, different questions come out: are all the accompanying domains essential for activity? Would the GH20 catalytic domain alone be functional? Which is the importance of GH20b? Several studies on the structure-function relationships at the active site have been performed [18–22] but only few have addressed the structural organization of these enzymes [23–25].

Regardless of the number of accompanying domains, we propose two different models of organization (Table 1): Model A, comprising proteins with at least both GH20b and GH20 domains, which is characterized by a GH20b-GH20-α architecture, where GH20 is always accompanied by GH20b at the N-terminus and followed by an extra α-helix after the GH20 (β/α)_8_-barrel, and Model B with proteins that only present the GH20 domain without GH20b.

**Fig 2:**
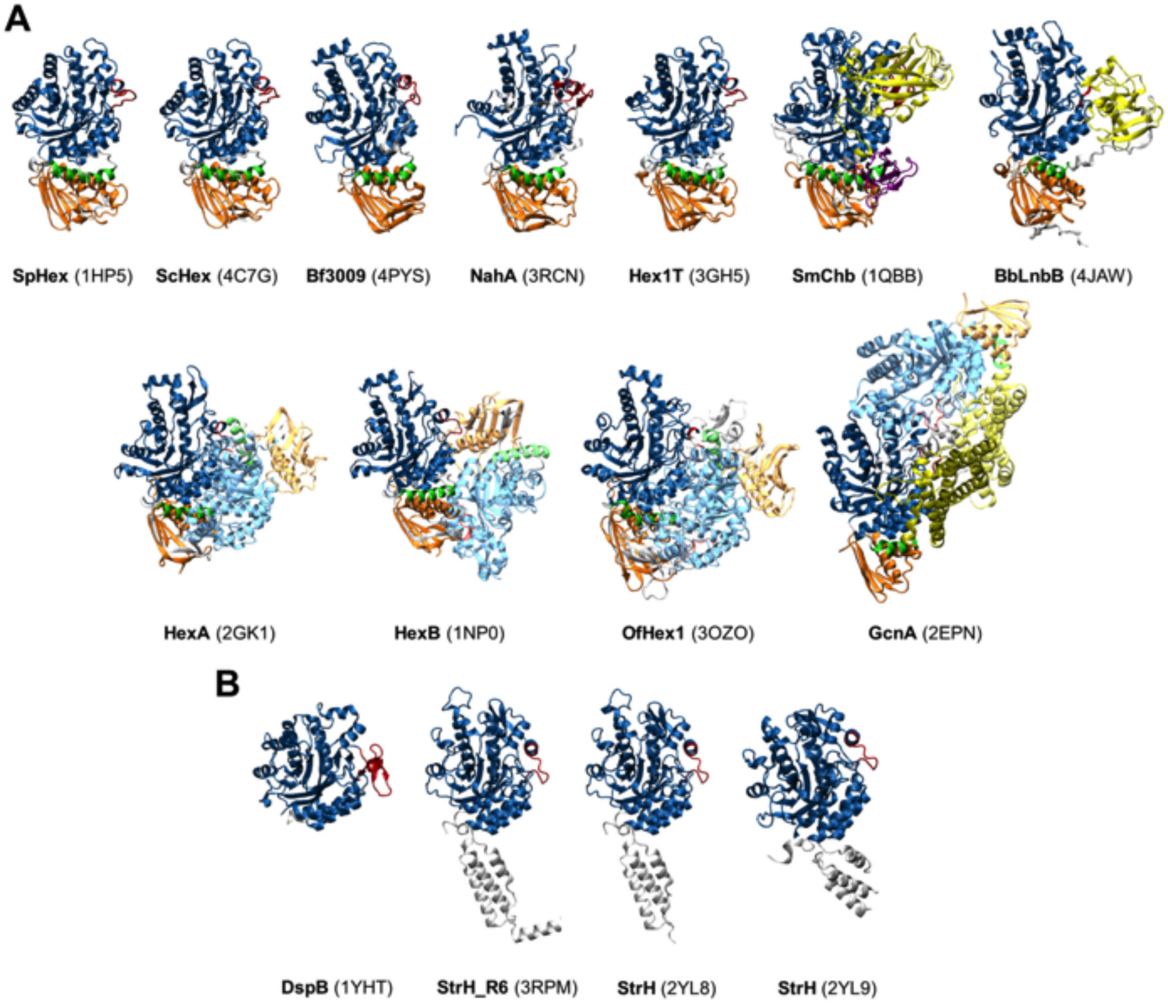
Crystal structures of GH20 β-N-acetylhexosaminidases. **(A) Structures according to model A architecture.** (B) Structures according to model B architecture. GH20b domains are colored in orange, GH20 domains in blue, α-helix in green, and the rest of accompanying domains in yellow and purple. Loop 2 from GH20 domain is in red. Dimer counterparts follow the same coloring scheme but with lower intensity.

To date, eleven of the thirteen GH20 enzymes structures fit into Model A (Fig 2A). As summarized in Table 1, five of them are monomeric proteins in solution (SpHex, ScHex, Hex1T, SmCHB, and LnbB), whereas other four are dimeric proteins (HexA, Hex B, OfHex1, and GcnA). The oligomerization state of the *Arthrobacter aurescens* (NahA) and *Bacteroides fragilis* (Bf3009) enzymes remains unknown.

For the monomeric proteins, SpHex and ScHex structures, which share 94% sequence identity, comprise the GH20b domain followed by the catalytic GH20 domain [26,27]. Hex1T consists of four domains, GH20b and GH20, and domains III and IV which are described as concanavalin A-like lectin and cellulose-binding domains respectively, according to Superfamily database (Supfam). The Hex1T crystal structure is a truncated form (residues 1–502), with only the GH20b and GH20 domains [25]. SmCHB is also constituted by four domains, where the N-terminal domain (CHB_HEX) is annotated as a carbohydrate binding module although its function remains unknown, and the C-terminal domain (CHB_HEX_C) as an immunoglobulin-like fold domain (Supfam). The central domains correspond to GH20b and GH20 domains [21]. LnbB is formed by five domains. Domains I and II are the GH20b and GH20 ones, Domain III (residues 546–700) is similar to a lectin domain according to a PSI-Blast search, Domain IV (784–932) has sequence homology to CBM32 (Supfam), and domain V (962–1041) is described as a Immunoglobulin-like domain in PFam. The recent crystal structure of this enzyme corresponds to a C-terminal truncated form (41–663) with the GH20b, GH20 and part of the lectin-like domain [23]. Finally, NahA and Bf3009, with unknown oligomerization states but included in the group of monomeric proteins in Table 1, present only the GH20b and GH20 domains.

For the dimeric proteins, each subunit of HexA, HexB [28,29] and OfHex1 [30] have only the two domains, GH20b and GH20, while the GcnA structure contains an additional domain III, which is predominantly α-helical and is reported to form a significant part of the dimer interface [31]. In general, these accompanying domains are non-catalytic domains involved in binding and substrate specificity but still the mechanism by which this occurs remains uncertain [32,33].

The remaining two GH20 enzymes present structures that are assigned to Model B (Fig 2B): dispersin B from *Aggregatibacter actinomycetemcomitans* (DspB) and the β-hexosaminidases from *Streptococcus pneumoniae* (StrH). Both are monomeric proteins in solution where DspB presents only one domain, the unique GH20 domain [34], and StrH consists of four domains, where the N-terminus is a tandem repeat of two GH20 domains, designated as GH20-1 and GH20-2, and the C-terminus is a tandem repeat of two domains tentatively designated as G5 with unknown function (Pfam). Truncated forms containing a single GH20-1 and GH20-2 domains of *S*. *pneumoniae* were crystallized [24]. The GH20-1 and GH20-2 domains are quite similar although specific features confer differential substrate specificities.

### 2. *B*. *bifidum* lacto-N-biosidase truncated forms

**Fig 3:**
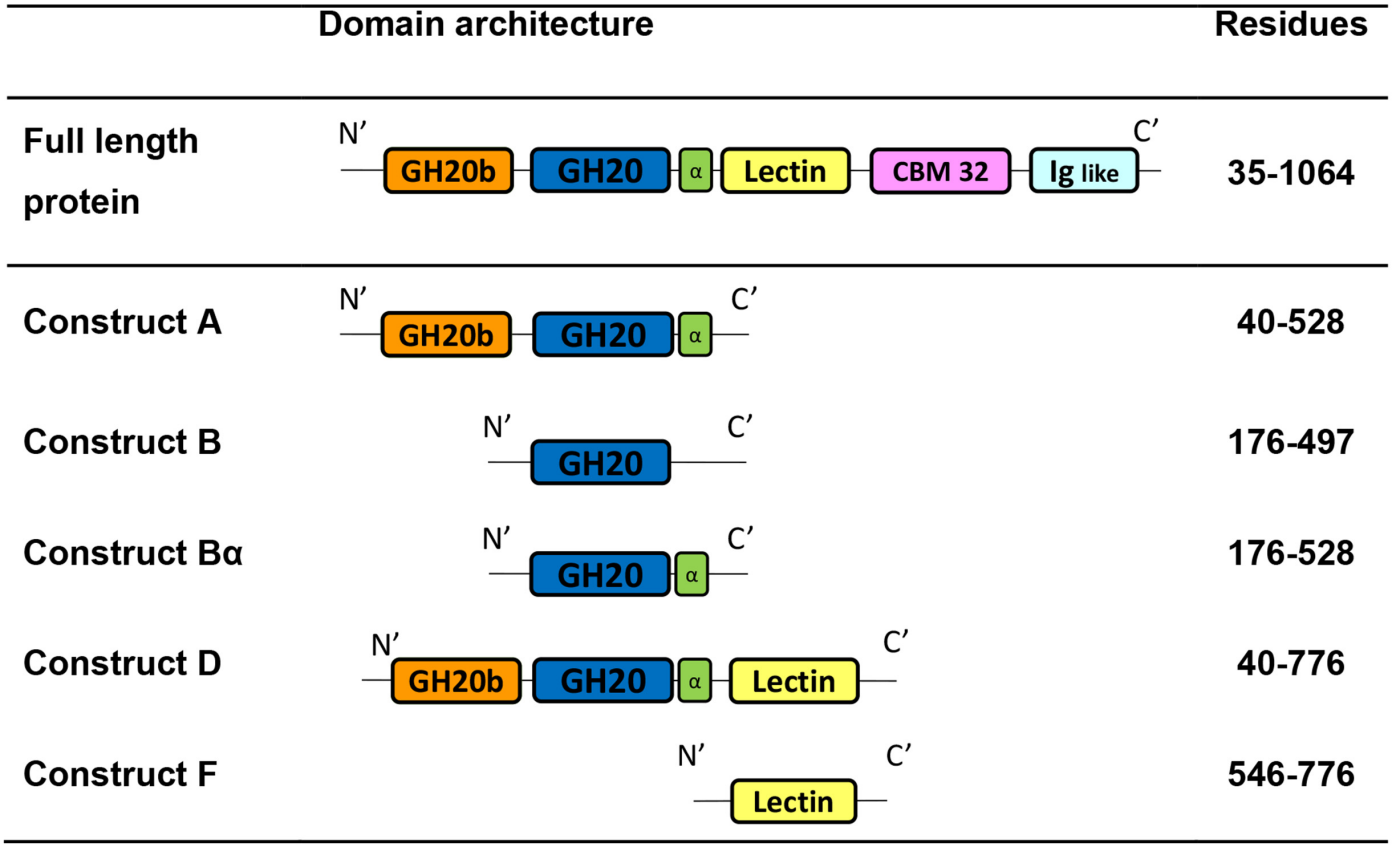
Truncated forms of *B*.*bifidum* Lacto-N-biosidase.

The full length protein was truncated according to the two different models of structure organization we propose, and three different truncated proteins were designed: construct A, B and Bα (Fig 3).

Construct A (residues 40–528) follows the architecture organization of Model A enzymes and is composed of the GH20b and GH20 domains always followed by the extra α-helix. This construct would be expected to be active since several enzymes of model A exhibit just these two domains alone in the wild-type protein (such as SpHex structure and HexA, HexB and OfHex1 subunits). Furthermore, a truncated form of Hex1T (encoding the GH20b-GH20-α domains alone) is reported to be active [25]. However, a previous work by Ito *et al*. (2013) in which different LnbB truncated forms were designed, revealed that a truncated protein (37–520) equivalent to construct A exhibits no catalytic activity. A longer truncated protein (37–663) composed by GH20b-GH20-α and a part of the lectin-like domain was reported to be as active as the full length. These data indicate that the GH20b-GH20-α architecture is not the minimal functional unit for LnbB, as it is for the rest of the model A GH20 enzymes. Further studies are presented to determine the details of the minimum requirement for LnbB to be catalytically active.

Construct B (residues 176–497) mimics Model B enzymes and is formed by the single catalytic GH20 domain alone. Lastly, construct Bα (residues 176–528) was designed to tackle the open question whether the accompanying GH20b domain can be removed in Model A enzymes. This construct encodes the catalytic GH20 domain with the extra α-helix at the C-terminus. Both constructs, B and Bα, were designed for the study of the behaviour of the GH20 catalytic domain in model A proteins.

**Fig 4:**
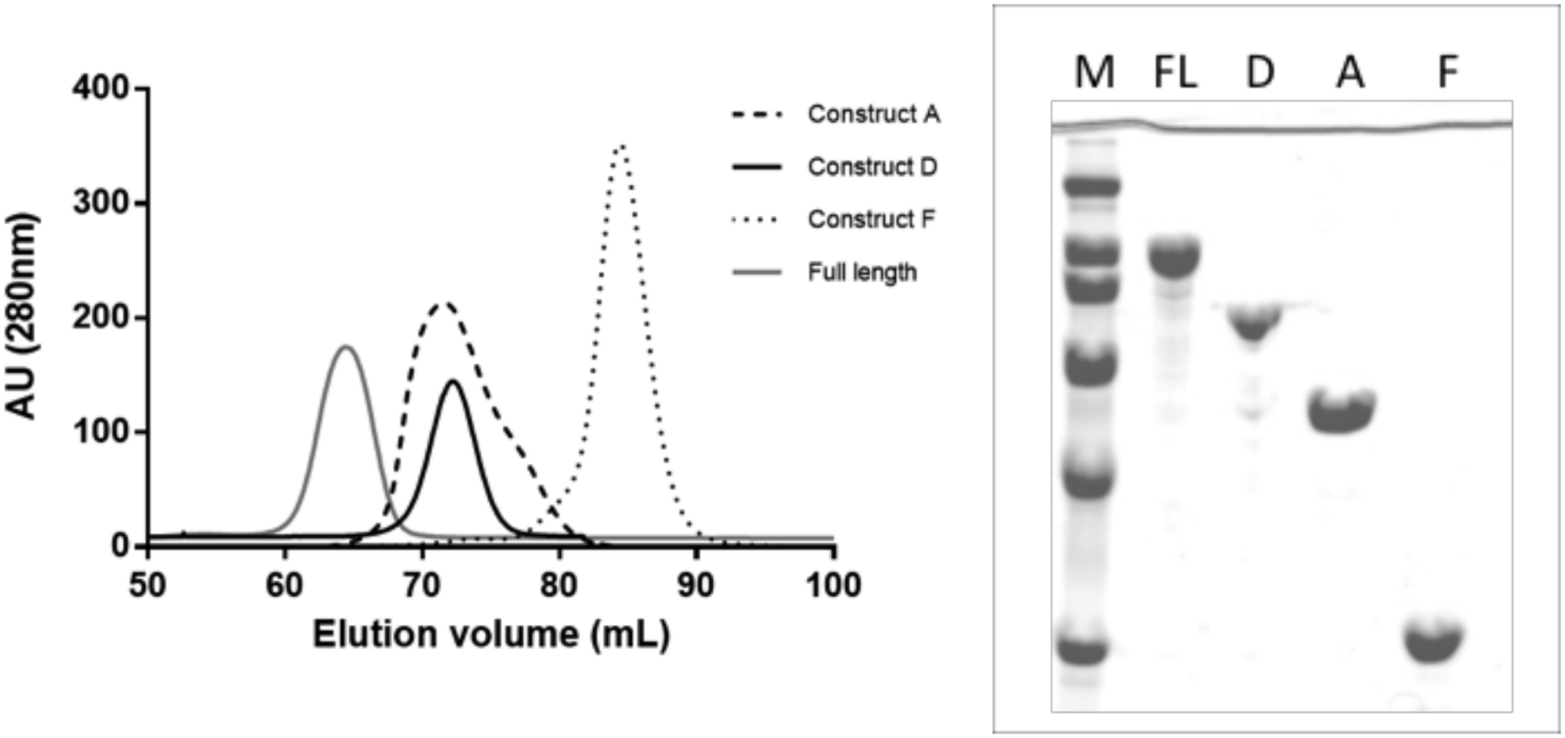
Size exclusion chromatography of the full length protein and different constructs of Lacto-N-biosidase from *B*.*bifidum*. (A) Chromatogram of the full length protein (FL) and constructs A, D and F. (B) SDS-PAGE of the eluted proteins. M: broad range protein molecular weight marker (200, 116.2, 97.4, 66.2, 45, 31 kDa).

Engineered proteins of LnbB enzyme were cloned and expressed in *E*. *coli*. The full length protein (37–1064), without the signal peptide and membrane anchor regions, was expressed as C-terminal His_6_-Tagged protein. Constructs A, B and Bα were expressed as N-terminal His_6_-Tagged proteins. Cultures were grown at 30°C for 24 h with autoinduction medium. The full length protein and construct A were obtained as soluble intracellular proteins with yields of 16 and 27 mg/L of culture respectively. SDS-PAGE analysis and MALDI-TOF mass spectrometry confirmed the theoretical 112 kDa of the full length protein and 57 kDa of Construct A. Further characterization by gel filtration chromatography showed that the full length protein is monomeric with particle size of about 10 nm by dynamic light scattering, whereas Construct A is mainly dimeric in solution (Fig 4). The full length protein was also expressed as N-terminal His_6_-Tagged protein to evaluate the effect of the N-terminal tag. Both, C-terminal and N-terminal His_6_-Tagged full length proteins, were soluble and had the same specific activity (data not shown).

On the other hand, constructs B and Bα were only present in the insoluble pellet and no soluble protein was achieved after different expression conditions such as autoinduction or IPTG induction at different temperatures (data not shown). This seems to indicate that the single GH20 catalytic domain of LnbB, with or without the extra α-helix, is not stable enough and yields insoluble aggregates. Thus, GH20b domain at the N-terminus of GH20 is required for LnbB expression and stability. This domain is present in all model A enzymes being the relative orientation of GH20b and GH20 catalytic domain the same in all structures (Fig 2). The number of β-strands of the GH20b domain varies (for example five in GcnA, six in HexA and HexB and seven in SpHEx and LnbB) but the interface between both domains is well conserved: two loops (residues 54–60 of loop 1 and residues 127–130 of loop 4 in LnbB) and three helices (residues 82–99, 146–163 and the extra α-helix of the GH20 domain which is oriented toward an hydrophobic pocket). GH20b domain is not only found in GH20 enzymes but also in some GH84 hexosaminidases and GH67 glucoronidases annotated in Pfam as GH20b and GH67N [35], respectively, always located at the N-terminus of the catalytic domain. Considering this scenario, we conclude that GH20b-GH20-α architecture of model A enzymes cannot be reduced to that of model B.

The hydrolytic activity of the soluble constructs was assayed using p-nitrophenyl β-lacto-N-bioside as substrate at pH 4.5 and 30°C. Full length protein showed a specific activity of 27.2 ± 3.5 s^-1^, but construct A was inactive, as expected according to previous studies [23]. The GH20b-GH20-α architecture of LnbB is enough to guarantee protein expression, but this architecture does not correspond to the minimal functional unit maybe requiring other accompanying domains from the C-terminus for the enzyme to be active.

### 3. Structural requirements in the GH20 domain for functionality

**Fig 5:**
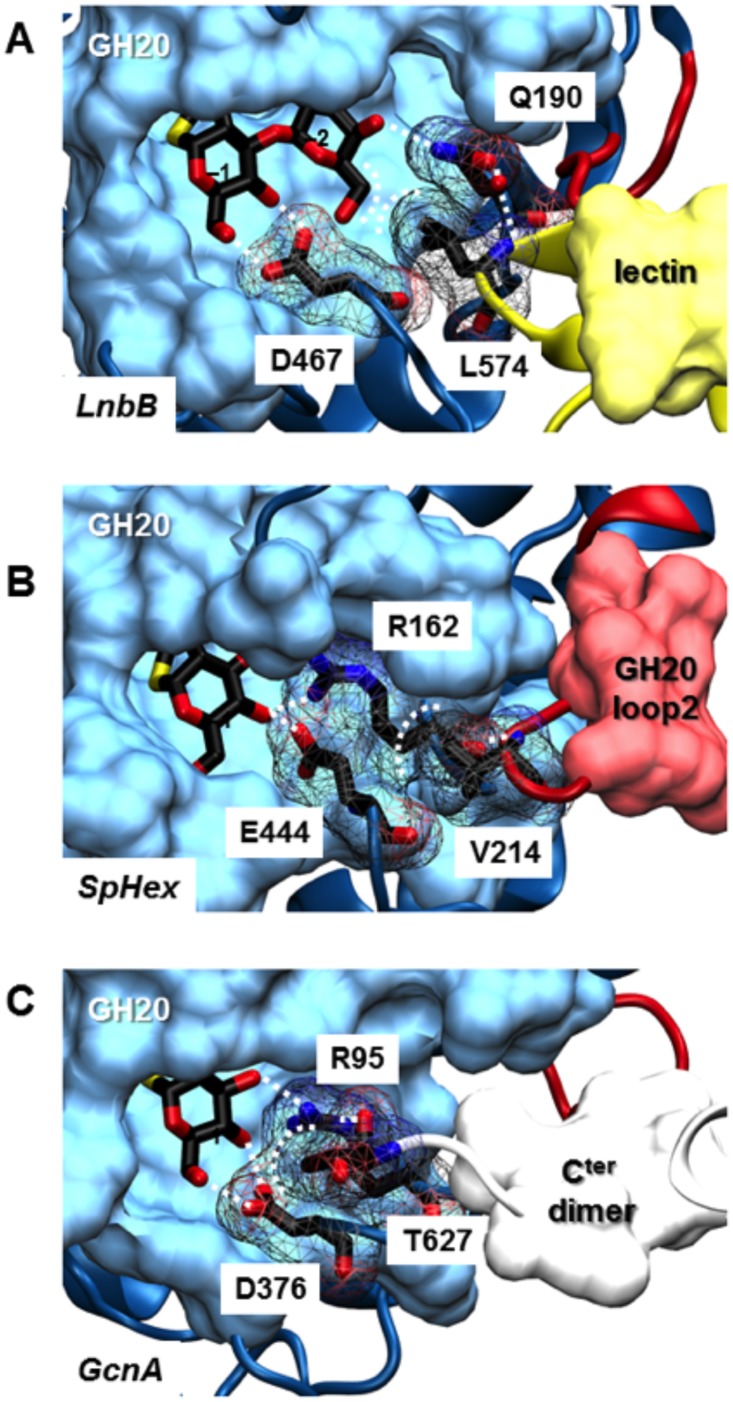
Remote elements and their key residues that complement the active site of GH20 β-N-acetylhexosaminidases. (A) *B*. *bifidum* lacto-N-biosidase. (B) *S*. *plicatus*. (C) *S*. *gordonii* N-acetylhexosaminidases. Dot lines represent interactions between residues of the remote element and the active site as indicated in Table 2.

While this minimal GH20b-GH20-α architecture assures the activity of SpHex and Hex1T [25,36] and also of dimeric proteins such as HexA, HexB and OfHex1 [30,37,38], it is not enough for LnbB. The crystal structure of LnbB (residues 41–663) in complex with the cyclic intermediate analogue lacto-N-biose-thiazoline [23] consisting of three out of the five domains (GH20b, GH20 and part of the lectin-like domain) was active. The accompanying lectin domain resulted to be the missing element required in our construct A to be fully active. We have carefully analyzed the interaction of the catalytic GH20 domain with other C-terminus domains in all crystallized protein structures of model A (Fig 5). Exploring the structure, important structural features can be assigned. The lectin domain interacts with the catalytic GH20 domain, providing a remote element that folds on the active site (Fig 5A). This element (residues 570–578) consists of a beta-turn, in which Leu574 completes the substrate binding cavity favouring a hydrophobic interaction with the hydroxymethyl group of the galactose moiety of the substrate. In addition, Leu574 is positioned between two conserved amino acids Gln190 and Asp467 of subsites -1 and -2 forming further interactions between this turn and the active site (Fig 5A). For instance, through a water mediated hydrogen bond, the backbone nitrogen atom of Leu574 favours the orientation of Gln190 towards the O4 of the galactose unit of the disaccharide ligand. Engineered forms of LnbB in which part of the C-terminus of the lectin domain were even shorter [23] or completely removed as in our construct A, showed an important decrease in activity of the enzyme, indicating that the interactions we observe in the structure between the remote lectin domain and the substrate binding site are essential for catalysis.

**Fig 6:**
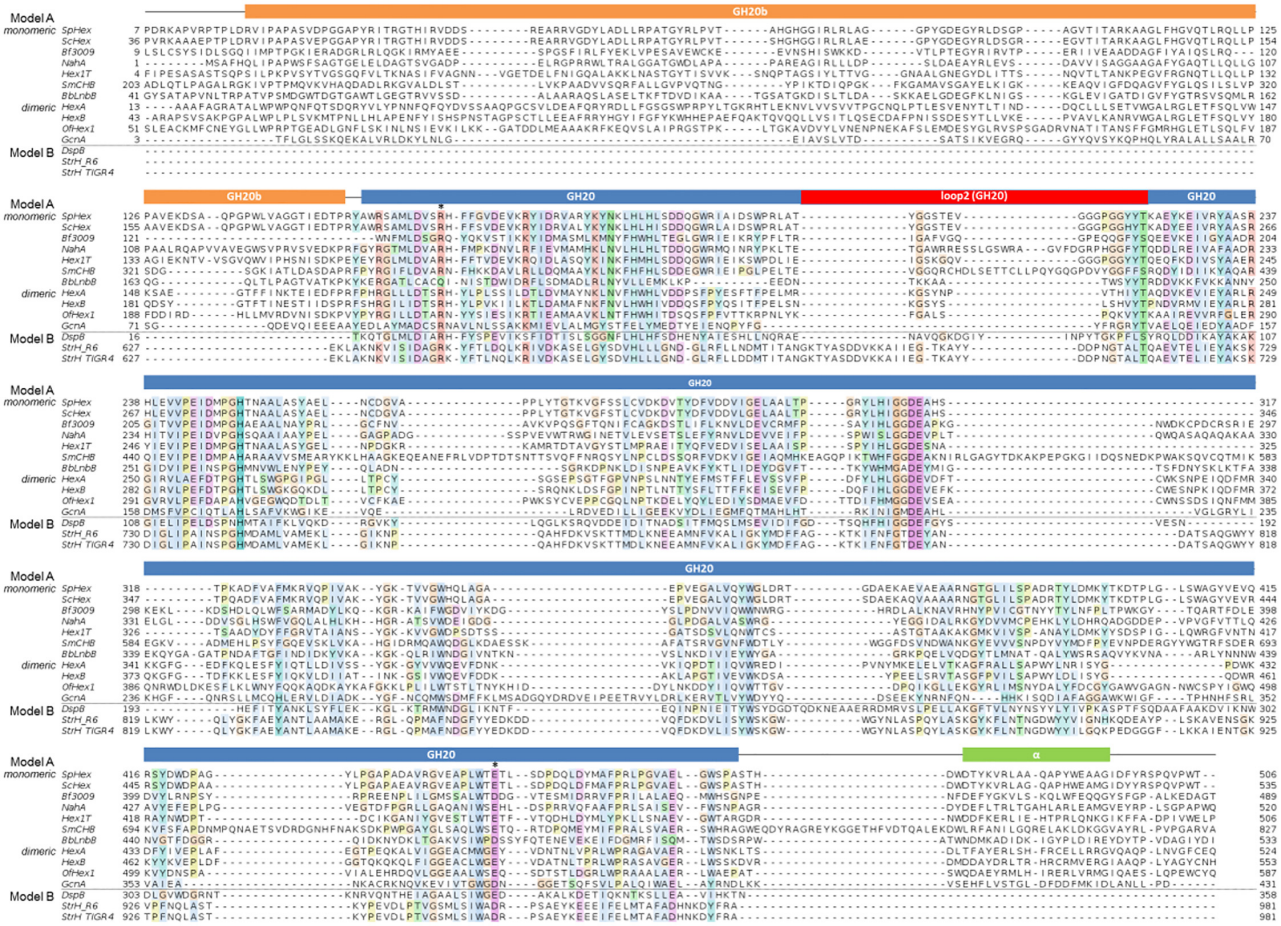
Multiple sequence alignment of β-N-acetylhexosaminidases of known structure from family GH20. Domains distribution is shown as colored boxes on top. Accompanying domains are not shown. Conserved arginine and glutamate/aspartate sites are marked with a star. Sequences were downloaded from UniProt entry names: SpHex (O85361_STRPL), ScHex (Q9L068), Bf3009 (Q5LAT3) NahA (A1RBZ5_ARTAT), Hex1T (D2KW09_9BACL), SmCHB (CHB_SERMA), LnbB (B3TLD6_BIFBI), HexA (HEXA_HUMAN), HexB (HEXB_HUMAN), OfHex1 (Q06GJ0_OSTFU), GcnA (Q6ST21_STRGN), DspB (Q840G9_AGGAC), StrH_R6 (Q8DRL6_STRR6), StrH_TIGR4 (STRH_STRPN). Alignment was performed with PROMALS [17]. Conserved positions are coloured according to ClustalW colour scheme.

Interestingly, we have detected that this pattern is reproduced in all proteins of model A: there is always a remote element assisting in the definition of the substrate binding cavity by means of direct interactions with conserved amino acids directly involved in protein—substrate interactions. Table 2 summarizes the exact localization of these remote elements for each member of model A and the interactions it forms with conserved active site residues. In general, this remote element is provided by a long loop of the GH20 domain itself (loop 2). In the cases where this loop is too short, the remote element comes from an accompanying domain (such as the lectin domain in the case of LnbB) or from the C-terminus of the other monomer in the case of dimeric proteins. In detail, structures of SpHex, ScHex, NahA, Bf3009, SmCHB and Hex1T present this remote element coming from the extended loop 2 of the GH20 domain itself (Fig 5B). This loop positions a hydrophobic residue, such as Val214 in SpHex (equivalent to Leu574 in LnbB), into the second shell of the subsite -1 cavity just behind the conserved arginine and aspartate in all known GH20 hexosaminidases, Arg 162 and Asp444 (equivalent to Gln190 and Asp467 in LnbB, see alignment in Fig 6). These amino acid residues can form hydrophobic interactions between their side chains and also, a hydrogen bond between the backbone NH group of Val214 and the carbonyl group of the backbone of Arg162 (Fig 5B). These interactions between residues of the remote element and the conserved arginine and aspartate of subsite -1, are present in all the structures with long loop 2 (Table 2). ScHex presents Val243 of loop2 next to Arg191 and Asp473 in the same way. NahA and SmCHB show the longest loops 2 as seen in the alignment. They provide the remote elements next to the corresponding arginines, Arg146 and Arg349, and aspartates, Asp456 and Asp739 respectively. One arginine of the remote element, Arg196 in NahA and Arg399 in SmCHB, is hydrogen-bonded to the backbone of the active site arginine. Similarly, Bf3009 presents Gln209 of loop2 next to Arg153 and Asp450. In Hex1T, Val222 from the remote element is positioned behind Arg170 and Asp443 and the backbone NH group of this valine donates a hydrogen bond to the arginine carbonyl oxygen atom. Interestingly, a truncated form of Hex1T with only the GH20b and GH20 domains has been reported to be active [25]. Consistently with our explanations, this truncated form does not affect the definition of the active site, because this construct keeps the long loop 2 in the GH20 domain.

**Table 2:** Remote element and conserved interactions with the active site of model A GH20 β-N-acteylhexosaminidases.

Enzyme	Loop 2 length	Remote element	GH20 conserved Arg/Asp or Glu at subsite −1	Remote element interactions with conserved Arg/Asp	
				non-polar interactions	polar interactions
SpHex	Long G209-T223	Loop 2	R162	V214	V214
			E444	V214	-
ScHex	Long G238-T252	Loop 2	R191	V243	V243
			E473	V243	-
NahA	Long G193-T219	Loop 2	R146	-	R196
			E456	-	-
Bf3009	Long G200-S214	Loop 2	R153	-	Q209
			D450	-	
Hex1T	Long G217-T231	Loop 2	R170	V222	V222
			E443	V222	-
SmCHB	Long G396-S425	Loop 2	R349	Y413	R399
			E739	Y413	-
LnbB	Short T231-T236	Lectin G570-T578	Q190*	L574	S233
			D467	L574	-
HexA	Short αG225-αT235	C-terminus subunit β A543-N552	αR178	βA548	βG549
			αE462	βY547	-
HexA	Short βG258-βT267	C-terminus subunit α A514-E523	βR211	αV519	αG520
			βE491	αV519	-
HexB	Short G258-T267	C-terminus dimer A514-E523	R211	A548	G549
			E491	Y547	-
OfHex1	Short G267-T276	C-terminus dimer A577-S594	R220	P582	E583
			E526	P582	-
GcnA	Short G136-T143	C-terminus dimer S622-T627	R95	-	T627
			D376	T627	-

*Residue in LnbB substituting the function of the conserved arginine but in subsite -2.

By contrast, when loop 2 is short, this remote element comes from the accompanying domains, as in LnbB, or from the C-terminus domains of the other monomer in dimeric structures, such as, GcnA, HexA, HexB and OfHex1. For example, in the homodimeric structure of GcnA, domain III forms a significant part of the dimer interface [31]. The C-terminus of domain III of chain B (622–627) winds back into the structure and contacts the catalytic GH20 of chain A (Fig 5C). Specifically, the C-terminus amino acid residue Thr627 projects into subsite -1 and it is adjacent to the conserved arginine and aspartate (Arg95, Asp376) and is part of the active-site pocket of the dimer partner. Besides, the carboxyl group of this C-terminal Thr627 and the Arg95 side chain are at less than 3 Å distance, establishing electrostatic interactions. As in LnbB, any truncated form of this C-terminus domain III is believed to be inactive. In the dimeric structures of HexA, HexB and OfHex1 [28–30], dimerization occurs exclusively between the GH20 catalytic domains. Dimerization is essential for catalysis since residues of one subunit structurally complete the active site of the other one. For example, Tyr456 of subunit β is part of the active site of subunit α in HexA. In addition, the C-terminus of subunit β provides this remote element very close to the subsite-binding cavity of subunit α and vice versa (Table 2). Particularly, the hydrophobic residue Ala548 (subunit β) of this element in HexA forms part of the second shell of the cavity behind the conserved Arg178 (subunit α) and Asp462 (subunit α), and the backbone nitrogen atom of Gly549 (subunit β) interacts by hydrogen bond with the carbonyl oxygen atom of Arg178 (subunit α). The equivalent interactions between subunits are also seen in HexB and OfHex1 crystal structures (Table 2).

Therefore, all GH20 structures of model A clearly show the presence of a remote element folding on the substrate-binding cavity providing amino acid residues that either complete the active site, as in the extended subsite -1 of the *S*. *gordonii* hexosaminidase or in subsite -2 of the LnbB, or participate in the second shell of this cavity behind the conserved arginine and aspartate residues in subsite -1 of hexosaminidases.

**Fig 7:**
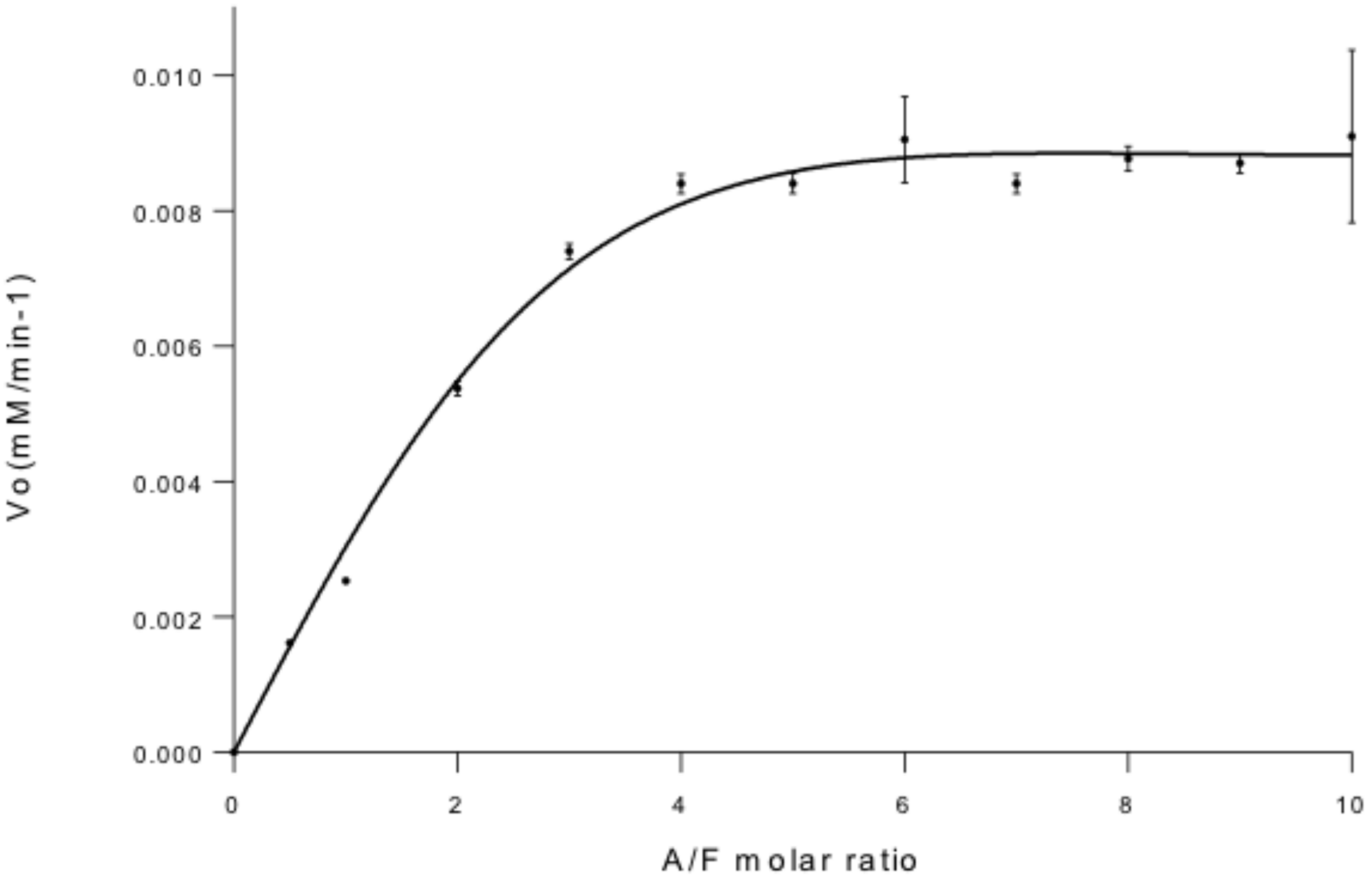
Complementation assay of construct A with construct F at different molar ratios. Conditions: 50 nM construct A, 25 nM-500 nM construct F, 0.25 mM p-nitrophenyl β-lacto-N-bioside, 25 mM citrate-25 mM phosphate buffer, pH 4.5, 30°C. The relative standard deviation did not overcome 2% except at 6 and 10 molar ratio that was lower than 15%.

**Fig 8:**
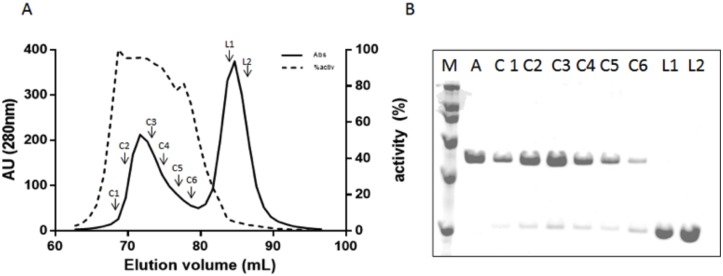
Size exclusion chromatography of the complementation assay in a ratio 1:6 of constructs A and F. (A) Chromatogram of the eluted fractions: absorbance at 280nm (-), hydrolase activity (•••). (B) SDS-PAGE of different fractions. M: broad range protein molecular weight marker (200, 116.2, 97.4, 66.2, 45, 31 kDa); A: construct A Standard, fractions C1–C6, fractions L1–L2. The relative standard deviations for the molecular weights and activities were less than 2% and 15% respectively.

To provide experimental evidence of the requirement of this remote element in LnbB, a complementation assay was designed. If Construct A (composed of the GH20b and GH20-α domains) is inactive because of the lack of the remote element, would addition of the lectin domain (as a single protein) reconstitute the active site and restore activity? Toward this end, Construct F (the single lectin domain, Fig 3) to assay complementation with Construct A, as well as Construct D (Fig 3), which contains the non-catalytic GH20b domain, the catalytic GH20 domain and the lectin domain, were expressed. The C-terminal His_6_-Tagged proteins were purified and analyzed by SDS-PAGE and MALDI-TOF, confirming the corresponding theoretical molecular masses of 83 and 28 kDa for constructs D and F, respectively. Gel filtration chromatography showed that both proteins were monomers in solution (Fig 4). Construct D was active with the same specific activity than the full length enzyme on p-nitrophenyl β-lactobioside (27 s^-1^). Therefore, both C-terminal domains in LnbB, the CBM32 and Ig like domains are not important for the catalytic activity, and construct D is the minimal functional unit of LnbB. Next, a complementation experiment was assayed by adding Construct F (the single lectin domain) on the inactive Construct A. Activity was restored in a concentration-dependent manner when increasing the concentrations of Construct F (Fig 7). At a molar ratio of 1:6 activity levels off at 11% of the hydrolytic activity of the full length enzyme. The interaction between constructs A and F at a 1:6 molar ratio was analyzed by gel filtration chromatography (Fig 8). Fractions L1–L2 correspond to the excess of Construct F, the lectin domain with molecular weight of 28 kDa, and fractions C1–C6 to proteins with molecular weight of 74 kDa that could be the dimer of construct A (as in Fig 4) and the complex between A and F with theoretical molecular mass of 85 kDa. The hydrolase activity of Fractions C1–C6 and the presence of lectin in these fractions seen in SDS-PAGE (Fig 8) demonstrate that the complex between A and F is formed and active. As it is observed in Fig 8, not all construct A protein is complexed with construct F. By densitometry analysis we determined that the lectin represents 10% of total protein in the mixture. Assuming a 1:1 interaction, 10% of Construct A is in complex with Construct F and therefore the specific activity of the complex is similar to that of the full length protein. To exclude non-specific interactions, complementation assay was performed with Construct A and BSA at different concentration ratios and no activity recovery was found (data not shown).

## Conclusions

This structural-functional analysis allows us to come to different conclusions. First, we identified two levels of domain organization in GH20 enzymes: model A with GH20b-GH20-α architecture and model B with the single catalytic GH20 domain alone. As LnbB constructs show, model A enzymes cannot be reduced to model B, since GH20b domain is a structural requirement in the N-terminus of GH20 to assure protein stability. Secondly, GH20b-GH20-α architecture is not always the minimal functional unit. For the LnbB enzyme here studied, the isolated GH20b-GH20-α domain is inactive and requires the lectin domain which provides an essential loop to shape the active site. Based on these results and analysis of the structures of GH20 enzymes, we propose a broader mechanism by which important interactions in the substrate binding cavity of the catalytic GH20 domain are provided by a remote element in all structures, which must be preserved to ensure a proper definition of the active site. The remote element can directly be provided by a long loop 2 of the GH20 domain itself. In this case, GH20b-GH20-α can act as a minimal functional unit, and large multidomain enzymes can be engineered to reduced forms with a minimal model A architecture. In other circumstances, when loop 2 is short, proteins dimerize or present an accompanying domain in the C-terminus of GH20 that provide this remote element. In the latter cases, protein engineering of these enzymes must be designed maintaining the accompanying domains. The proposed model can stimulate further studies on other members of the superfamily.
